# Surgical Reconstruction to Allow Endovascular Access for Flow Diversion of Giant Cavernous Aneurysm: A Combined Approach

**DOI:** 10.7759/cureus.2381

**Published:** 2018-03-28

**Authors:** Visish M Srinivasan, Ascher Kaufmann, Peter Kan, Edward A Duckworth

**Affiliations:** 1 Department of Neurosurgery, Baylor College of Medicine; 2 Department of Neurosurgery, St. Lukes Regional Medical Center

**Keywords:** giant aneurysm, flow diversion, hunterian ligation, carotid endarterectomy, cerebral aneurysm, cervical aneurysm

## Abstract

Giant cavernous aneurysms of the internal carotid artery (ICA) are challenging lesions associated with high surgical morbidity. Prior to the past several years, these were treated by surgical reconstruction, proximal ligation, or stent-assisted coiling techniques. Flow diversion has become the standard of care for these lesions, providing a high rate of obliteration with a much better safety profile. However, flow diverters rely upon a navigable vasculature and, usually, a tri-axial support system. Cases in which such access is difficult require unique approaches to combine the strengths of both surgical and endovascular therapy. A woman with a giant cavernous ICA aneurysm and an ophthalmic artery aneurysm presented for treatment, but access was challenging due to cervical ICA tortuosity and pseudoaneurysms. We elected a staged, combined approach with surgical reconstruction of the cervical ICA followed by flow diverter placement for the intracranial aneurysms. Our case features an “outside-the-box” approach that synergistically applied both microsurgical and endovascular techniques to treat a challenging pathology. Classic microsurgical techniques remain important in cases that are refractory or not amenable to endovascular therapy alone.

## Introduction

Prior to the development of modern neurointerventional surgery, giant cavernous aneurysms presented a treatment challenge; surgery was associated with high technical difficulty and morbidity and endovascular options were limited. The introduction of flow-diverting devices, such as the pipeline embolization device, represents a paradigm shift in the treatment of such lesions [1]. However, flow diversion is not without caveats. It requires dual antiplatelet therapy (DAPT) prior to treatment, which is a contraindication in cases of ruptured aneurysm/intracranial hemorrhages [2]. Additionally, flow-diverting stents, due to their morphology and construction, are generally stiff and require strong (preferably) tri-axial systems for controlled deployment. Therefore, unlike relatively simpler neuroendovascular procedures, such as coiling, flow diversion (FD) requires a more navigable vasculature to provide appropriate support for these stiff devices [3].

When transfemoral or transradial endovascular access to an aneurysm is difficult to navigate or when the aneurysm itself is too challenging or dangerous for treatment by surgery or an endovascular treatment alone, open surgical and endovascular procedures can be employed for a combined approach [4]. “Comprehensive” cerebrovascular neurosurgeons with training and expertise in both modalities are uniquely positioned to address such aneurysms [5]. We present such a case that lent itself to a combined approach, where we used surgical techniques that facilitated endovascular access for two intracranial aneurysms, including a giant cavernous aneurysm.

## Case presentation

A 38-year-old female presented after being involved in a motor vehicle crash. The accident was initially considered secondary to a presumed stroke, as she reported an acute-onset severe headache and left-sided weakness. Her past medical history was significant for hypertension, long-standing retro-orbital headache, and a remote history of another motor vehicle crash.

**Figure 1:**
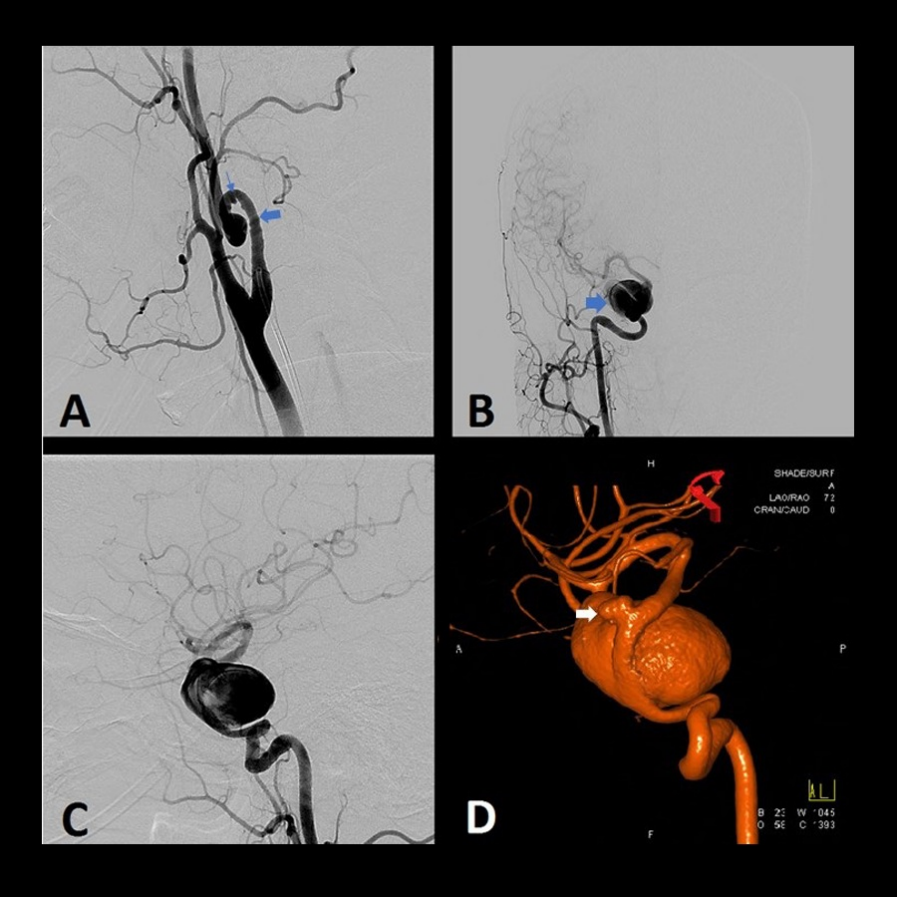
Preoperative angiography of the right common carotid artery showing cervical and intracranial aneurysms Preoperative angiography of the right common carotid artery showing cervical and intracranial aneurysms. (A) Lateral – cervical, (B) anteroposterior – head, (C) Lateral – head, (D) three-dimensional (3D) reconstruction. The cervical loop (large arrow) and pseudoaneurysms (thin arrows) precluded endovascular access to the giant cavernous (large blue arrow) and smaller ophthalmic aneurysm (white arrow).

Our work-up did not reveal evidence of ischemia. However, diagnostic angiography demonstrated bilateral highly tortuous internal carotid arteries (ICAs), with the right ICA harboring three pseudoaneurysms, the largest of which measured 8 x 7.7 mm (Figure 1A). Imaging also revealed a 3.5 x 2.5 cm fusiform giant cavernous aneurysm of the right ICA (Figures 1B-D). An ophthalmic artery aneurysm measuring 4.9 x 3 mm, distal to her fusiform giant aneurysm, was also noted (Figure 1D). The etiology and chronicity of her proximal pseudoaneurysms were unknown since no previous imaging was available to know when they arose. Potential causes included her longstanding severe hypertension, her remote trauma history, or both, but a definitive diagnosis could not be made.

Based on the patient’s young age, severe hypertension, ipsilateral retro-orbital headaches, and the possibility that her giant aneurysm had caused a transient ischemic attack (TIA), we felt justified to recommend treatment for her intracranial aneurysms. A balloon occlusion test was performed in anticipation of multiple scenarios, including a possible ICA sacrifice via Hunterian ligation. She passed clinically and based on nuclear medicine testing. However, due to the patient’s young age and the tenuous nature of collateral flow following vessel sacrifice, the multidisciplinary treating team was hesitant about such a deconstructive approach, especially as it would preclude any future endovascular treatments on that side.

In our case, the only feasible open approach for the giant cavernous aneurysm would have been trapping and bypass. This procedure would have carried the attendant risks and morbidity associated with extracranial-intracranial bypass, would have required two separate incisions, and would not necessarily address her multiple cervical pseudoaneurysms. One obvious consideration for the treatment of the patient’s two intracranial aneurysms was flow diversion. On the other hand, an endovascular-only approach would require navigating through the highly tortuous ICA and past the pseudoaneurysms. Any injury to the cervical ICA would have been difficult to rectify with stenting. The 360-degree loop and three pseudoaneurysms in the cervical ICA, compounded by tortuosity of the cavernous ICA proximal to the giant aneurysm, precluded safe access for endovascular intervention. Specifically, flow diverters are best delivered with a microcatheter within an intermediate catheter in a strong tri-axial support system [3].

Therefore, we instead opted for the surgical correction of the ICA, which would improve access to the fusiform giant cavernous aneurysm as well as the ophthalmic aneurysm.

Part 1: Surgical treatment of tortuous ICA

**Figure 2:**
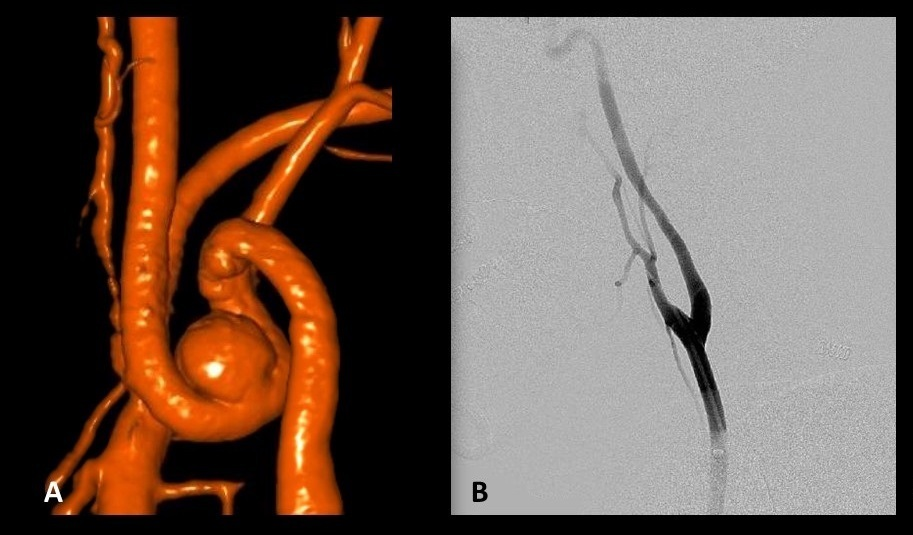
Comparison of preoperative and postoperative angiograms of the right cervical internal carotid artery Comparison of preoperative and postoperative angiograms of the right cervical internal carotid artery. (A) Three-dimensional (3D) angiogram showing a 360-degree loop with three pseudoaneurysms. (B) Postoperative right common carotid artery angiogram, showing patent reanastomosis and favorable, straight cervical segment for endovascular access.

**Figure 3:**
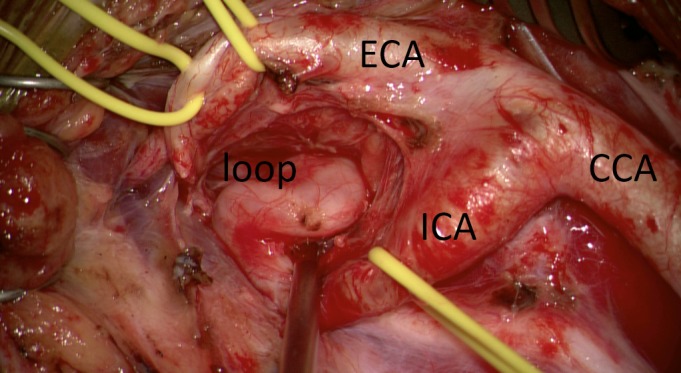
Intraoperative photograph of right cervical exposure The external carotid artery (ECA), common carotid artery (CCA), internal carotid artery (ICA), and excised pseudoaneurysm-containing loop are labeled. Note that the exposure extends more rostally than that used for a typical carotid endarterectomy.

In order to remove the tortuous anatomy and the pseudoaneurysms limiting definitive endovascular treatment, we decided the initial step in the patient’s therapy would be a “loopectomy,” with trapping and resection of the pseudoaneurysm and tortuous carotid loop followed by end-to-end reanastomosis of the shortened ICA (Figure 2). Surgery was performed via an oblique incision similar to a carotid endarterectomy (CEA) except with a bias toward a more superior exposure of the ICA. The large pseudoaneurysms and the redundant loop were identified and circumferentially dissected out (Figure 3). The diseased segment was trapped, resected, and an end-to-end anastomosis was performed (Video 1). Surgery was performed without complications, and the patient was discharged with a plan to return for delayed, definitive endovascular treatment of the intracranial aneurysms.

**Video 1:** Surgical reconstruction of the cervical internal carotid artery Via a standard cervical exposure, the proximal cervical internal carotid artery was exposed, including the tortuous loop containing several pseudoaneurysms. The loop was excised and an end-to-end anastomosis performed.

**Figure 4:**
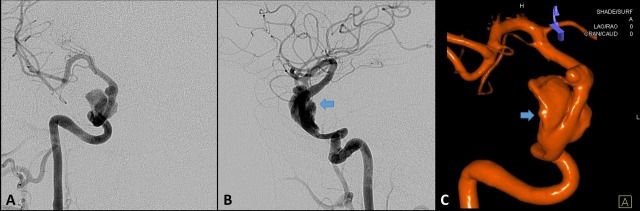
Postoperative angiography via right common carotid artery two weeks after surgical reconstruction Postoperative angiography via right common carotid artery two weeks after surgical reconstruction. Partial thrombosis (Hunterian ligation phenomenon) of the giant cavernous aneurysm following temporary trapping for the surgical resection of the carotid loop is seen in (A) anteroposterior view, (B) lateral view, and (C) three-dimensional (3D) reconstruction. Arrows indicate the scalloping of the aneurysm, with contrast opacifying the patent portion of the lumen, whereas the thrombus is not visible.

The patient was readmitted 13 days postoperatively with retro-orbital headaches. Diagnostic angiography demonstrated the patency of the operative end-to-end anastomosis site with a focal, non-flow-limiting stenosis at the site of anastomosis, as well as partial thrombosis of the fusiform giant aneurysm of the right cavernous carotid (Figure 4). The patient was encouraged to adhere to antiplatelet therapy and, after an uneventful hospitalization, was again discharged home in stable condition.

Part 2: Endovascular treatment

**Figure 5:**
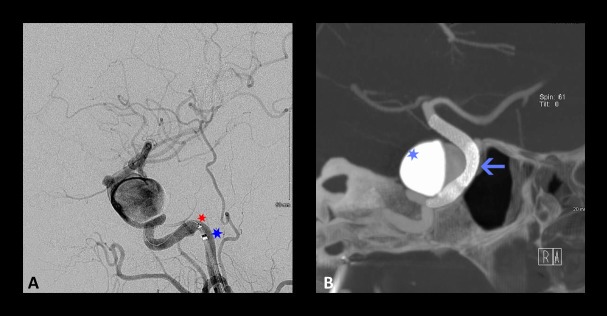
Intraprocedural angiography for the placement of a flow diverter Intraprocedural angiography for the placement of a flow diverter. (A) Lateral angiography showing the positioning of the intermediate catheter (blue star) and the microcatheter (red star) as part of a tri-axial catheter system and (B) Cone-beam computed tomography (CBCT) (Dyna CT) showing the positioning of two partially overlapping devices (arrow) between the distal petrous segment and the communicating segment of the ICA. Contrast stagnation can be appreciated within the aneurysm (light blue star).

After an uneventful three months at home, she returned for the endovascular treatment of her giant cavernous and smaller ophthalmic aneurysm by utilizing two overlapping flow diverters [6]. On the day of treatment, the ICA cervical segment was fully remodeled and no longer stenotic (Figure 5A). Also, the cavernous aneurysm had fully recanalized and was no longer thrombosed (Figure 5A). The now straight cervical ICA allowed us to employ a tri-axial catheter configuration comprising a 6-French Cook Shuttle (Cook Inc., Bloomington, Indiana, US) positioned in the cervical ICA, a Navien 058 intermediate catheter (Medtronic, Minneapolis, Minnesota, US) positioned in the cavernous ICA, and a 027 Marksman microcatheter (Medtronic, Minneapolis, Minnesota, US) initially positioned in the middle cerebral artery (MCA) (Figure 5A). The first of the two 3 x 25 mm flow diverters was advanced over a Synchro-10 microwire (Stryker Neurovascular, Fremont, CA, US) and deployed from the proximal communicating segment to a landing zone in the distal cavernous segment. The second flow diverter was deployed to overlap approximately 50% of the area covered by the first flow diverter and ended 1 cm proximal to the cavernous aneurysm. A progressive stagnation of flow within the cavernous aneurysm was noted with the placement of both devices. Cone-beam computed tomography (CBCT) was performed, which demonstrated good apposition of the flow diverters within the ICA (Figure 5B). There were no immediate technical or clinical complications of our intervention, although the patient was once again briefly readmitted on post-procedure day four with headaches and diplopia due to thrombosis of the giant aneurysm. However, she responded well to optimization of her pain medication.

Outcome

**Figure 6:**
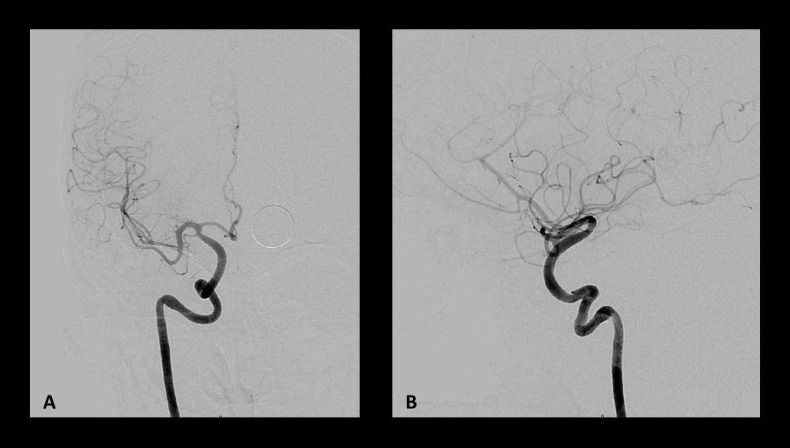
Postoperative angiography via right common carotid artery Postoperative angiography via right common carotid artery showing the obliteration of both intracranial aneurysms in (A) anteroposterior and (B) lateral view. The flow diverters span the distal petrous segment to the communicating segment of the internal carotid artery (ICA).

At the most recent follow-up (six months following flow diverter placement), the patient demonstrated no focal neurological deficits, and diagnostic angiography showed complete occlusion of both the giant cavernous and ophthalmic aneurysms (Figure 6).

## Discussion

We present the case of a patient whose pathology was not ideally treated by either surgical or endovascular techniques alone. The patient’s primary pathology was a giant aneurysm of the cavernous ICA, which was best treated through endovascular flow diversion by modern treatment paradigms [1,3,7]. However, the anatomy of the patient’s access vessel, a severely tortuous ICA with three pseudoaneurysms, precluded safe endovascular access to the lesion of interest. Instead, the optimal clinical outcome was achieved with the judicious application of both open and endovascular techniques [8]. The open stage included a standard cerebrovascular technique (a modified CEA). The endovascular stage, made safer by the preceding surgery, allowed the treatment of the index lesion by the standard-of-care technique [1,3,9]. If the patient had been treated with either microsurgical or endovascular techniques alone, she likely would have received sub-optimal therapy or been subjected to complications.

A Hunterian ligation effect was also evident in our case, illustrated by serial angiography (Figure 1, Figure 4). In 1785, Dr. John Hunter adopted his eponymous technique by ligating the external carotid artery of a stag. Initially, this produced a cold antler, but over time, it reperfused and continued to grow. At autopsy, he discovered that collateral circulation had developed in response to his intervention. Although attempts to treat aneurysms throughout the body had been recorded since the third century, the popularization of the Hunterian ligation, with the placement of only a proximal ligature, achieved a measure of consistently successful outcomes for the first time [2,6]. This principle led Victor Horsley to perform the first successful ICA sacrifice for the treatment of an aneurysm in 1885 [2].

Similarly, at two weeks postoperative of the patient’s “loopectomy” procedure, her giant cavernous aneurysm was largely thrombosed, presumably due to temporary trapping of the cervical ICA during surgery. However, as subsequent angiograms demonstrated, this effect was temporary, and the aneurysms were recanalized by the time the patient returned for definitive endovascular treatment (Figure 5). The patient was not yet on dual antiplatelet therapy (DAPT) during this time since the flow diversion procedure was scheduled at three-months post-loopectomy. Luckily, no thromboembolic complications occurred as a result of this partial thrombosis. Early postoperative antiplatelet therapy may be important to consider in similar cases in the future. Hemorrhagic risk due to the presence of a giant aneurysm while on antiplatelet therapy should be weighed against thromboembolic risk.

The use of hybrid operating rooms has become in vogue in cerebrovascular neurosurgery [10]. This combined approach was staged, in contrast to a true, one-stage “hybrid” operation done in a hybrid operating room. In this case, we elected for a staged operation to allow for the healing of the anastomosis. Had the surgical approach been done for access and not for the resection of the loop entirely, a single stage procedure may have been feasible.

Our case highlights that neurosurgeons, or multidisciplinary surgical teams, must maintain and employ a variety of open and endovascular skills. The skilled application of both interventional modalities uniquely allows the comprehensive cerebrovascular neurosurgeon to remain unencumbered by the limitations of their training, enabling him or her the freedom to select and apply the most appropriate therapeutic intervention to achieve the best possible outcome for their patients.

## Conclusions

Our case features an “outside-the-box” approach that synergistically applied both microsurgical and endovascular techniques to treat a challenging pathology. Classic microsurgical techniques remain important in cases that are refractory or not amenable to endovascular therapy alone.
